# Self-Burdensomeness, Self-Esteem and Suicidal Ideation

**DOI:** 10.1007/s10608-024-10477-x

**Published:** 2024-02-28

**Authors:** Tobias Teismann, Thomas E. Joiner, Morgan Robison, Julia Brailovskaia

**Affiliations:** 1 Mental Health Research and Treatment Center, Department of Psychology, Ruhr-Universität Bochum, Bochum, Germany; 2 Department of Psychology, Florida State University, Tallahassee, USA; 3 DZPG (German Center for Mental Health), partner site Bochum/Marburg, Marburg, Germany

**Keywords:** Suicidal ideation, Self-burdensomeness, Self-esteem, Interpersonal theory of suicide, Acute suicidal affective disturbance

## Abstract

**Background:**

Low self-esteem and self-burdensomeness have been proposed as risk factors for suicidal ideation. Yet, self-burdensomeness may be more relevant to suicidal ideation than low self-esteem. The purpose of the present study was to investigate the association between self-esteem, self-burdensomeness, and suicidal ideation in a sample of adult outpatients.

**Methods:**

Data from *N* = 202 patients (66.3% female; age: *M*[*SD*] = 39.87 [13.31], range: 19–73) who started therapy at an outpatient clinic were collected. A subsample of *n* = 111 patients (68.5% female; age: *M*[*SD*] = 38.50 [13.48], range: 20–73) also took part in a second assessment three-months later.

**Results:**

Self-burdensomeness was shown to predict suicidal ideation concurrently and prospectively – after controlling for age, gender, depression, and self-esteem. Furthermore, self-burdensomeness completely mediated the association between self-esteem and suicidal ideation. However, the reverse relationship, where the association between self-burdensomeness and suicidal ideation is mediated by self-esteem, was not supported.

**Conclusions:**

Self-burdensomeness might be understood as a driver of suicidal ideation. Findings point to the possibility that a focus on self-burdensomeness and/or low self-esteem might be relevant in the treatment of suicidal patients.

## Introduction

Self-esteem refers to an individual’s subjective evaluation of their worth as a person (Leary & Baumeister, 2000), which includes aspects of self-acceptance, self-satisfaction, and self-respect. Low self-esteem, in turn, is characterized by a dysfunctional and destructive self-evaluation. Low self-esteem is highly prevalent in clinical samples (Silverstone & Salsali, 2003) and has been shown to be a risk factor for both suicidal ideation and suicide attempts (Buecker et al., 2023; Soto-Sanz et al., 2019). Furthermore, studies have shown that self-disgust and self-hate – as an extreme expression of low self-esteem – are associated with suicide risk (e.g., Brake et al., 2017; Turnell et al., 2019). Still, most people suffering from low self-esteem will neither contemplate suicide nor attempt suicide (Buecker et al., 2023; Teismann et al., 2019). Therefore, it seems necessary to explore mechanisms that might underlie the association between low self-esteem and suicidal ideation.

In the context of the *Interpersonal Theory of Suicide* (ITS; Joiner, 2005; Van Orden et al., 2010), perceived burdensomeness, i.e., the view that one’s existence is a burden on family members and friends (“I am a burden to others”), has been proposed to be a proximal and a causal risk factor for suicidal ideation. In fact, there is ample evidence that perceived burdensomeness is associated both with low self-esteem (e.g., Eades et al., 2019; Van Orden et al., 2012; Zhornitsky et al., 2020) and with suicidal ideation (Chu et al., 2017; Ma et al., 2016). The association between perceived burdensomeness and suicidal ideation has been observed in different samples (e.g., students, out- and inpatients), using different assessment strategies, and while controlling for concurrent risk factors (e.g., age, gender, depression, hopelessness; Hill & Pettit, 2014). Additionally, perceived burdensomeness has been found to predict suicidal ideation and behavior in longitudinal studies (e.g., Forkmann et al., 2020; Rath et al., 2021). Consequently, perceived burdensomeness has been included as one feature of an acute suicidal syndrome, the Acute Suicidal Affective Disturbance (ASAD) syndrome (Rogers et al., 2023; Stanley et al., 2016). ASAD is proposed to be an acute arousal state defined by the four criteria: “(a) A geometric increase in suicidal intent over the course of hours or days (as opposed to weeks or months); (b) One (or both) of the following: marked social alienation (e.g., severe social withdrawal, disgust with others, perception that one is a liability on others), and/or marked self-alienation (e.g., views that one’s selfhood is a burden, self-disgust); (c) Perceptions that the foregoing are hopelessly intractable; and (d) Two or more manifestations of overarousal (e.g., insomnia, nightmares, agitation, irritability)” (Stanley et al., 2016, p. 98). The importance of being a burden to others is displayed in criterion B. However, criterion B does not only highlight the relevance of perceiving oneself as a burden to others (*other-burdensomeness*), but also the relevance of perceiving one’s selfhood as a burden (*self-burdensomeness*). Self-burdensomeness refers to the view that one is not only flawed and deficient but that these painful feelings and thoughts about the self are intolerable (“I can`t stand myself”, “I can`t stand being aware of myself”). It is as if the self’s continuance – that is, existence – is an intolerably heavy weight to bear. As a consequence, a person might anticipate that the nothingness of death would feel better than the distress of living with those intolerably burdening self-feelings. In line with this, suicide has been contextualized as an escape from aversive self-awareness (Baumeister, 1990).

In a first study, self-burdensomeness has been shown to be associated with perceived burdensomeness as well as suicidal ideation (Teismann et al., 2023); it was furthermore shown that other-burdensomeness mediated the influence of self-burdensomeness on suicidal ideation and vice versa, suggesting that feeling like a burden to others can internalize to the self and elicit suicidal thoughts. Alternatively, feeling like a burden to oneself might contribute to the perception of being a burden to others, leading to suicidal ideation. However, self-burdensomeness has rarely been studied, and it remains particularly unclear to what extent self-burdensomeness is related to low self-esteem. One might speculate that low self-esteem could fuel a perception of being a burden to oneself and that self-burdensomeness might mediate the association between low self-esteem and suicidal ideation.

Given this background, the purpose of the present study was to investigate the association between self-esteem, self-burdensomeness, and suicidal ideation in a sample of adult outpatients. More specifically, this study examined if (1) self-burdensomeness is predictive of suicidal ideation (after controlling for other risk factors including self-esteem) and, if (2) self-burdensomeness mediates the association between self-esteem and suicidal ideation. We hypothesize that self-burdensomeness will be a stronger predictor of suicidal ideation than low self-esteem; furthermore, we hypothesize that self-burdensomeness will fully mediate the association between self-esteem and suicidal ideation.

## Methods

### Participants

The total sample comprises *N* = 202 patients (66.3% female; age: *M*[*SD*] = 39.87 [13.31], range: 19–73; 100% Caucasian) who started cognitive-behavioral therapy at an outpatient university clinic in the Ruhr-area in Germany between April 2015 and October 2015 and completed a pretreatment data assessment prior to their intake (T1). A subsample of *n* = 111 patients (68.5% female; age: *M*[*SD*] = 38.50 [13.48], range: 20–73) also took part in a second assessment after 12 therapy sessions (T2; *M* = 3.27 month, [*SD* = 0.92] after the pretreatment assessment). Of note, the survey was terminated by the study organizers after approximately 100 patients had taken part in the second survey; it is therefore not the case that the other patients discontinued treatment and/or participation in the study of their own accord. A multivariate analysis of variance (MANOVA), Hoteling’s trace T = 0.050, *F*(6, 195) = 1.621, *p* = .143, revealed that participants who took part in both assessments did not significantly differ from the other participants regarding age, *F*(1, 200) = 2.635, *p* = .106, gender, *F*(1, 200) = 0.498, *p* = .481, suicidal ideation, *F*(1, 200) = 0.931, *p* = .336, self-esteem, *F*(1, 200) = 0.437, *p* = .509, and self-burdensomeness, *F*(1, 200) = 0.945, *p* = .332. However, patients who did not complete the follow-up assessment had higher depression scores at baseline than patients who completed the follow-up assessment, *F*(1, 200) = 6.190, *p* = .014. The most common primary diagnoses according to the International Classification of Diseases (ICD-10) were Recurrent Depressive Disorder (21.6%), Single Episode Depressive Disorder (11.0%), Panic Disorder with Agoraphobia (9.6%), Social Phobia (6.9%), and Posttraumatic Stress Disorder (5.5%). In total, 52.5% (*n* = 106) reported current suicidal ideation (DSI-SS≥1) and 9.4% (*n* = 19) reported at least one lifetime suicide attempt.

All patients were informed that the clinic regularly conducts research and provided informed consent prior to participation. In order to assure a standard of quality, all clients seeking help at the clinic are required to fill out questionnaires prior to their intake. No compensation is given to clients for doing so. This study was reviewed and approved by the local Ethics Committee.

### Measures

#### Rosenberg Self-Esteem Scale (RSES; Von Collani & Herzberg, 2003)

Self-esteem was assessed using the 10-item RSES (e.g. “On the whole, I am satisfied with myself”). Items are rated on a 7-point Likert-type scale ranging from 1 (strongly disagree) to 7 (strongly agree), with higher values indicating stronger self-esteem. In a German sample, the measure showed good Cronbach`s α as well as convergent and discriminant validity (von Collani & Herzberg, 2003). Internal consistency in this sample was *α* = 0.92.

#### Self-Burdensomeness Scale (SBS; Gebauer et al., 2009)

Self-burdensomeness was assessed with a modified version of the Perceived Burdensomeness (PB) Subscale of the Interpersonal Needs Questionnaire (INQ; Van Orden et al., 2012). As such, in the six items of the INQ-PB the term “people in my life” became replaced by “I/myself.” For example, the item “These days I think I make things worse for the people in my life” was changed to “These days I have been making my own life situation worse.” Accordingly, the SBS assesses perceptions of self-burdensomeness with six items that are to be answered on a 7-point Likert-type scale ranging from 1 (not at all true for me) to 7 (very true for me). Higher values indicate that the respondent believes they are a burden to themselves. An exploratory factor analysis (EFA) using the Maximum Likelihood method (ML; rotation method: promax) revealed a unidimensional factor structure for the six items used in the present study, Kaiser-Meyer-Olkin = 0.849; Barlett’s test of sphericity: χ^2^ = 1377,278, df = 15, *p* < .001; eigenvalue of the factor: 4.351; explained variance of the factor: 72.5%; factor loads range: 0.693 – 0.956. The SBS has been shown to have adequate internal consistency (*α* = 0.92) and to significantly correlate with the Perceived Burdensomeness-Subscale of the INQ (*r* = .73, *p* < .001) and with suicidal ideation (*r* = .41, *p* < .001; Teismann et al., 2023). Accordingly, internal consistency was good in the current study: *α* = 0.94.

#### Depressive Symptom Inventory – Suicidality Subscale (DSI-SS; Joiner et al., 2002; German Version: Von Glischinski et al., 2016)

The DSI-SS is a 4-item scale designed to measure the frequency and intensity of suicidal ideation symptoms over the past two weeks. Scores on each item range from 0 (e.g., “I do not have thoughts of killing myself”) to 3 (e.g., “I always have thoughts of killing myself”), with higher values indicating more severe suicidal ideation. The German version of the scale has been shown to possess strong psychometric properties (von Glischinski et al., 2016). The internal consistency (Cronbach’s *α*) for the DSI-SS in the current sample was *α* = 0.92.

#### Suicide Behavior Questionnaire – Revised (SBQ-R; Osman et al., 2001; German Version: Glaesmer et al., 2018)

The SBQ-R comprises four items assessing different aspects of suicidal behavior (lifetime suicidal ideation, suicide plans, suicide attempts, 12-month suicidal ideation, suicidal communication, and one’s estimation of the probability of a future suicide attempt). Each item utilizes a different Likert scale with a sum score of 18 points indicating the highest severity of suicidal behavior. Psychometric properties of the SBQ-R are well-established (Osman et al., 2001; Glaesmer et al., 2018). In the present study, only responses to the question about lifetime suicide attempts (Item 1: “I have attempted to kill myself, and really hoped to die”) were analyzed.

#### Depression-Anxiety-Stress Scales 21 – Depression Subscale (DASS-21D; Henry & Crawford, 2005)

The DASS-21D comprises seven items asking about depression symptoms within the past week (e.g., “I could not seem to experience any positive feeling at all”). Items are rated on a 4-point Likert-type scale (0 = did not apply to me at all; 3 = applied to me very much or most of the time), with higher values indicating more severe depressive symptomatology. Psychometric properties of the DASS-21 are well established in both clinical and non-clinical studies (Henry & Crawford, 2005). Internal consistency in the current sample was *α* = 0.92.

### Statistical Analyses

Statistical analyses were conducted using SPSS 28 and the macro Process version 4.0 (www.processmacro.org/index.html; Hayes, 2021). Descriptive analyses were conducted, and the relationships between the investigated variables were assessed by zero-order bivariate correlation analyses. To evaluate the relationship between self-burdensomeness and suicidal ideation, two 3-step hierarchical regression analyses were conducted with self-reported suicidal ideation as the dependent variable – using T1 suicidal ideation in one of the analyses (Model 1) and T2 suicidal ideation in the other (Model 2). Age, gender, and depression were entered in Step 1, baseline self-burdensomeness was entered in Step 2, and baseline self-esteem was entered in Step 3. Cohen’s *f*^2^ served as effect size measure. Both models were not threatened by multicollinearity (all values of tolerance > .25; all variance inflation factor values < 5; Urban & Mayerl, 2006). Finally, a statistical mediation model (Process: model 4) was calculated using data from the prospective study sample. The model included self-esteem (T1) as a predictor, self-burdensomeness (T1) as a mediator and suicidal ideation (T2) as the outcome, controlling for age, gender, and depression (T1) as covariates. The basic relationship between self-esteem and suicidal ideation was denoted by *c* (total effect). The path of self-esteem to self-burdensomeness was denoted by *a*; *b* denoted the path of self-burdensomeness to suicidal ideation. The indirect effect (*ab*) was represented by the combined effect of path *a* and path *b*. Path *c’* denoted the direct effect of self-esteem to suicidal ideation after the inclusion of self-burdensomeness into the model. The bootstrapping procedure (10,000 samples) that provides percentile confidence intervals (95% CI) assessed the mediation effect. In addition, a “reversed” mediation model was calculated with self-burdensomeness (T1) as predictor and self-esteem (T1) as mediator to further analyze the hypothesized directional relation between self-esteem, self-burdensomeness and suicidal ideation.

To calculate the smallest required sample size, we conducted power analyses using the G*Power program, version 3.1 (Faul et al., 2009), for correlations and regression analyses, and the work of Fritz and MacKinnon (2007) for mediation analyses. The power analyses for mediation analyses (power > 0.80, *α* = 0.05, effect size: *f*^*2*^ = 0.15; cf., Mayr et al., 2007) required the largest samples size (*N* = 78). Thus, our sample size is sufficient for valid results.

## Results

Table 1 shows descriptive statistics and correlations of the assessed variables. The correlation analyses revealed that self-esteem (T1) was significantly negatively correlated with self-burdensomeness (T1; large effect), depression (T1; large effect), and suicidal ideation (T1, T2; both: moderate effect). Self-burdensomeness (T1) was significantly positively correlated with depression (T1; large effect) and suicidal ideation (T1, T2; both: large effects). Depression (T1) was significantly positively correlated with suicidal ideation (T1: large effect; T2: moderate effect). In addition, suicidal ideation (T1) was significantly positively correlated with suicidal ideation (T2; large effect).

### Concurrent Associations between Self-Burdensomeness, Self-Esteem, and Suicidal Ideation

In Step 1 of Model 1, *F*(3,198) = 25.137, *p* < .001, effect size *f*^2^ = 0.36 (large effect), depression was significantly positively associated with suicidal ideation, whereas age and gender did not show significant effects. In Step 2, *F*(4,197) = 29.090, *p* < .001, *f*^2^ = 0.56 (large effect), depression and self-burdensomeness were significantly positively associated with suicidal ideation; age and gender did not show significant effects. In Step 3, *F*(5,197) = 23.656, *p* < .001, *f*^2^ = 0.56 (large effect), again depression and self-burdensomeness showed a significant effect; age, gender and self-esteem were not significantly associated with suicidal ideation (see Table 2). The pattern of results did not change when excluding depression as a predictor variable.

### Prospective Associations Between Self-Burdensomeness, Self-Esteem, and Suicidal Ideation

In Step 1 of Model 2, *F*(3,107) = 7.735, *p* < .001, *f*^2^ = 0.18 (moderate effect), baseline depression was significantly positively associated with suicidal ideation (T2), and age and gender did not show significant effects (see Table 2). In Step 2, *F*(4,106) = 9.642, *p* < .001, *f*^2^ = 0.31 (moderate effect), only baseline self-burdensomeness was significantly positively associated with suicidal ideation (T2); age, gender, and depression did not show significant effects. In Step 3, *F*(5,105) = 7.647, *p* < .001, *f*^2^ = 0.30 (moderate effect), again only baseline self-burdensomeness was significantly positively associated with suicidal ideation; age, gender, depression and baseline self-esteem did not show significant effects. The pattern of results did not change when excluding depression as a predictor variable.

### Self-Burdensomeness as a Mediator Between Self-Esteem and Suicidal Ideation

**Fig. 1:**
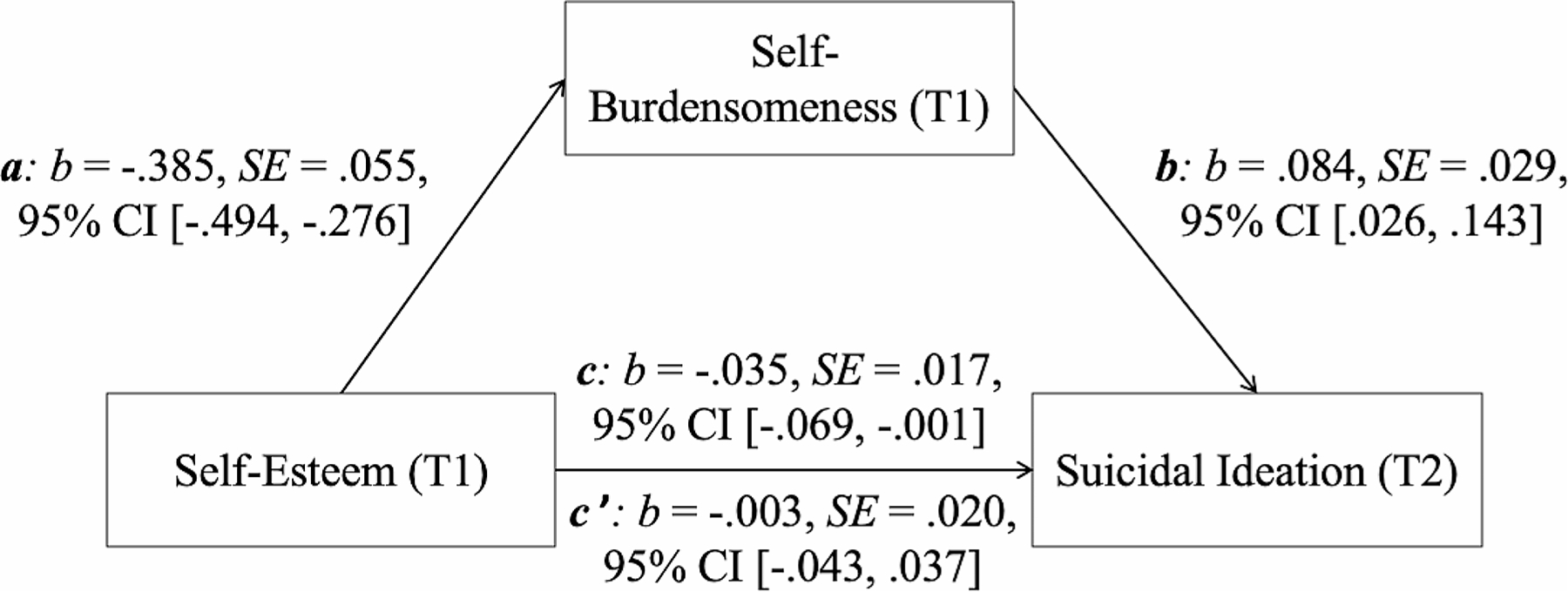
Mediation model including self-esteem (T1, predictor), self-burdensomeness (T1, mediator), and suicidal ideation (T2, outcome). *Note*. *c* = total effect, *c*’=direct effect; *b* = standardized regression coefficient, SE = standard error, CI = confidence interval

The statistical mediation model that included self-esteem as a predictor, self-burdensomeness as a mediator and suicidal ideation as the outcome was significant (see Fig. 1). Self-burdensomeness (T1) significantly mediated the negative association between self-esteem (T1) and suicidal ideation (T2). The basic link between self-esteem and suicidal ideation was significant (total effect, *c*: *p* = .043). The relationship between self-esteem and self-burdensomeness (path *a*: *p* < .001), and the link between self-burdensomeness and suicidal ideation (path *b*: *p* = .005), were also significant. The relationship between self-esteem and suicidal ideation was no longer significant after the inclusion of self-burden-someness in the model (direct effect, *c’*: *p* = .887). The indirect effect was significant (*ab*: *b* = − 0.032, SE = 0.013, 95% CI [−0.061, − 0.009]).

Notably, the “reverse” mediation model that included self-burdensomeness as a predictor, self-esteem as a mediator and suicidal ideation as the outcome was not significant (indirect effect: *ab*: *b* = 0.002, SE = 0.018, 95% CI [−0.033, 0.037]).

## Discussion

The aim of the present study was to examine the association between self-esteem, self-burdensomeness, and suicidal ideation. There were two main findings: (1) Self-burdensomeness concurrently and prospectively predicted suicidal ideation – even after controlling for age, gender, depression, and self-esteem. (2) Self-burdensomeness completely mediated the association between self-esteem and suicidal ideation.

In line with a previous study (Teismann et al., 2023), the current findings point to the possibility that self-burdensomeness could be a risk factor for suicidal ideation. It is not yet possible to make a statement on the relative importance of self-burdensomeness compared to other-burdensomeness. While there is much empirical support for the importance of perceived burdensomeness (Chu et al., 2017; Ma et al., 2016), this is only the second study to examine and demonstrate the importance of self-burdensomeness as a potential driver of suicidal ideation (cf., Teismann et al., 2023). In consequence, it is not yet clear whether the equal mention of self- and other-burdensomeness in the description of the Acute Suicidal Affective Disturbance (ASAD) syndrome (Rogers et al., 2023; Stanley et al., 2016) is justified. Nonetheless, the current findings represent a starting point for further and more specific investigations of the ASAD syndrome.

The present study also contributes to the literature on the relationship between self-esteem and suicidal ideation (Buecker et al., 2023; Soto-Sanz et al., 2019). In this regard, self-burdensomeness was shown to be highly correlated with self-esteem, yet, more strongly associated with suicidal ideation than low self-esteem. Furthermore, self-burdensomeness completely mediated the association between self-esteem and suicidal ideation, pointing to the possibiliy that low self-esteem is relevant to suicidal ideation only when it condenses into self-burdensomeness. The reverse relationship, where an association between self-burdensomeness and suicidal ideation is mediated by self-esteem was not supported, suggesting that the relationship between self-esteem and suicidal ideation is explained by perceptions of self-burdensomeness. A key difference between self-burdensomeness and low self-esteem may lie in the *intolerability* of negative self-views. According to the understanding proposed here, such intolerability is a hallmark feature of self-burdensomeness (see also Baumeister, 1990), but intolerability is no part of low self-esteem. A second key difference might be a strong notion of *self-blame* inherent in self-burdensomeness. People who suffer from self-burdensomeness do not only experience intolerably burdening self-threat, but they potentially believe that the causes of those painful thoughts and feelings, result from their very own wrongdoings. Future studies should further validate self-burdensomeness through convergent and divergent associations with neighboring constructs (e.g., self-blame, self-hate, and self-disgust) that have previously been associated with suicidal ideation (Brake et al., 2017; Turnell et al., 2019). Such investigations will more clearly delineate which type of negative self-evaluation is particularly relevant for suicidal ideation.

Clinically, the present results point to the possibility that a focus on low self-esteem and self-burdensomeness in the treatment of suicidal patients could be relevant (at least for some suicidal individuals). Various treatment programs are available for the treatment of low self-esteem (Chmielewski & Hanning, 2021; Fennell, 2022), which have proven to be effective in initial studies (Kolubinski et al., 2018; Niveau et al., 2021) - even with regard to an amelioration of suicidal ideation (Dat et al., 2022). Yet, to date, for the specific treatment of self-burdensomeness, there is a lack of treatment recommendations. Teismann et al. (2023) have suggested to use techniques to foster self-compassion, purpose and meaning making, two-chair dialogues focusing on the “inner critic” as well as strategies for dealing with negative emotionality to reduce the impression of being a burden on oneself (cf., Gordon, 2021; Lieberman et al., 2023). However, additional evidence for the importance of self-burdensomeness as a driver of suicidal ideation is needed before testing and implementing specific interventions aimed at reducing self-burdensomeness.

Several limitations must be considered when interpreting the results of the present study. First, since the sample was 100% Caucasian, it is unclear how the findings would generalize to more diverse populations. Furthermore, future studies should be conducted using an inpatient sample with more intense suicidal ideation and behavior. Second, in the regression analysis focusing on T2 suicidal ideation (Model 2), T1 suicidal ideation was not used as a predictor. In principle, prior suicidal ideation is a strong predictor of subsequent suicidal ideation (Franklin et al., 2017), Teismann et al., (2023) showed that self-burdensomeness is not predictive of future suicidal ideation over and above baseline suicidal ideation. At the same time, it does not seem reasonable to control for prior suicidal ideation in all models if, at the same time, one wishes to identify “suicide drivers” (cf., Jobes, 2023) that may represent a therapeutic starting point for changing suicidal ideation. Third, the Rosenberg scale used in the current study made use of a 7-point Likert-type scale instead of a 4-point Likert-type scale. This was due to a technical error. Still, the results of the current study are unaffected by the differing scale format, yet sum scores are not directly comparable to other studies. Fourth, psychometric properties of the Self-Burdensomeness Scale (Gebauer et al., 2009) have not been tested in an independent investigation. This should be done in a future study. As part of a validation study, correlations with related measures (e.g., self-hate, self-blame, mental pain) should also be examined. Fifth, due to the fact that self-esteem and self-burdensomeness were measured at the same timepoint, the current study can only provide a test of statistical mediation. A longitudinal study with at least three assessments is needed to provide a more thorough test of mediation. Finally, only part of the sample took part in the post-treatment assessment. This was for reasons inherent to the study; at the same time, it cannot be ruled out that this could have resulted in a systematic bias, especially as there were differences in baseline depression scores between participants who took or took not part in the posttreatment assessment. Accordingly, replications of the current findings are important before far-reaching conclusions can be drawn.

To conclude, the current study provides further evidence on self-burdensomeness, a state of aversive self-awareness, in understanding suicidal ideation in an outpatient sample. Furthermore, the study adds to the growing body of evidence relating self-esteem to suicidality.

## Supplementary Material

supplemental

## Figures and Tables

**Table 1:** Descriptive statistics and bivariate correlations of the investigated variables

	M(SD)	2)	3)	4)	5)
1) Self-Esteem (T1)	35.30 (15.70)	− 0.772**	− 0.681**	− 0.445**	− 0.426**
2) Self-Burdensomeness (T1)	20.48 (10.96)		0.688**	0.591**	0.506**
3) Depression (T1)	10.32 (5.94)			0.514**	0.404**
4) Suicidal Ideation (T1)	1.60 (2.20)				0.695**
5) Suicidal Ideation (T2)	1.25 (2.09)				

*Notes*. *N*_T1_=202, *n*_T2_=111; *M* = Mean; *SD* = Standard Deviation;

** *p* < .001

* *p* < .05

**Table 2:** Hierarchical regression analyses predicting suicidal ideation (T1, Model 1and T2, Model 2)

	Model 1, *N* = 202					Model 2, *n* = 111				
	*ß*	95%*CI*	T	Adjusted *R^2^*	Changes in *R*^*2*^	*ß*	95%*CI*	T	Adjusted *R^2^*	Changes in *R*^*2*^
*Step 1*				0.265	0.276				0.155	0.178
Age (T1)	− 0.107	[− 0.038, 0.002]	−1.768			− 0.115	[− 0.045, 0.009]	−1.314		
Gender (T1)	0.015	[− 0.487, 0.623]	0.243			− 0.045	[− 0.980, 0.580]	− 0.508		
Depression (T1)	0.519**	[0.148, 0.237]	8.569			0.409**	[0.088, 0.217]	4.658		
*Step 2*				0.359	0.096				0.239	0.089
Age (T1)	− 0.073	[− 0.033, 0.009]	−1.157			− 0.021	[− 0.030, 0.024]	− 0.245		
Gender (T1)	0.015	[− 0.451, 0.586]	0.257			− 0.058	[−1.001, 0.481]	− 0.383		
Depression (T1)	0.205*	[0.017, 0.135]	2.551			0.121	[− 0.040, 0.131]	1.045		
Self-Burdensomeness (T1)	0.449**	[0.058, 0.123]	5.471			0.424**	[0.039, 0.135]	3.578		
*Step 3*				0.360	0.005				0.232	0.000
Age (T1)	− 0.017	[− 0.023, 0.017]	− 0.288			− 0.017	[− 0.031, 0.026]	− 0.185		
Gender (T1)	0.010	[− 0.471, 0.567]	0.183			− 0.057	[−1.003, 0.494]	− 0.674		
Depression (T1)	0.244*	[0.028, 0.154]	2.835			0.115	[− 0.050, 0.135]	0.915		
Self-Burdensomeness (T1)	0.512**	[0.065, 0.141]	5.325			0.413**	[0.026, 0.143]	2.867		
Self-Esteem (T1)	0.120	[− 0.010, 0.043]	1.257			− 0.021	[− 0.043, 0.037]	− 0.142		

*Notes*. *ß*=standardized coefficient beta; *CI* = confidence interval;

** *p* < .01

* *p* < .05
