# Supplementary material for: Self-Burdensomeness, Self-Esteem and Suicidal Ideation
Source: Cognit Ther Res. Author manuscript; Available in PMC 2025 Aug 19. (PMC12360707; doi:10.1007/s10608-024-10477-x)
Supplement: supplemental [file NIHMS2049027-supplement-supplemental.docx]

**Acknowledgements**

The authors would like to thank Jochen Gebauer for his idea to differentiate self-budensomeness from other-burdensomeness.

**Statements and Declarations**

Competing interests: none.

Declarations of interest: none.

This research did not receive any specific grant from funding agencies in the public, commercial, or not-for-profit sectors.

**Credit author statement**

**Tobias Teismann:** Writing – original draft and revised version, coordinated the study, performed participant recruitment, data collection, data processing and statistical analysis. **Thomas Joiner:** Writing – original draft and revised version, interpretation of the data. **Morgan Robison:** Writing – original draft and revised versions. **Julia Brailovskaia:** Writing and statistical analysis. All authors read and approved the final version of the manuscript.
